# The largest secure corridor of the infra-acetabular screw—a 3-D axial perspective analysis

**DOI:** 10.1186/s12891-021-04433-z

**Published:** 2021-06-16

**Authors:** Bei Zhao, Wei Zhang, Hao Li, Liren Han, Shizhang Han, Xiaofei Yang, Jun Yan, Weidong Mu

**Affiliations:** 1 Department of Orthopaedics, Liaocheng People’s Hospital, Liaocheng, Shandong China; 2 Department of Traumatic Orthopaedics, Shandong Provincial Hospital Affiliated to Shandong First Medical University, Jinan, Shandong China

**Keywords:** Acetabular fracture, Axial perspective, Positional screw, Digital measurement

## Abstract

**Background:**

The infra-acetabular screw which is placed from the pubis to the ischium can be used as a special positional screw of the posterior column of the acetabulum. This study was performed to simulate the surgical procedure and obtain the ideal insertion point, diameter, length and angle of the screw through the method of axial perspective in Chinese patients.

**Methods:**

We randomly collected the pelvic computed tomography (CT) scans of 200 adults. DICOM-formatted CT-scan images were imported into Mimics software to establish the 3D digital model of the right semi-pelvic was established. A virtual cylinder representing the screw was placed from the pubis to the ischium to fix the posterior column. The largest secure diameter and length of the virtual screw were measured and the position of the insertion point and the directions of the screw were also researched.

**Results:**

The screw insertion safe zone exhibits an irregular shape of “tear drop” in the reconstructed pelvic model. The mean maximum diameter of screws was 5.01 ± 1.28 mm, and the mean maximum length of screws was 93.99 ± 8.92 mm. The screw insertion corridor with the least diameter 3.5 mm was found in 94 of 100 males (94%) and 86 of 100 females (86%). We found gender-dependent differences for the mean maximum diameter and the maximum length of the screw. There was statistically significant difference between genders in the position of insertion point.

**Conclusions:**

In this study, we suggest an individual preoperative 3D reconstruction simulation to develop better screw placement plans, which provides a valuable guideline for seeking the largest secure corridor of infra-acetabular screw. Further biomechanical studies are needed to verify the function of the screw.

## Background

The treatment of complex acetabular fractures involving quadrilateral displacement has been widely studied in recent years [1–6]. The goal of acetabular surgery is perfect reduction and rigid fixation, allowing early joint movement and avoiding postoperative complications [1, 5, 7].

Early, Letournel and Judet suggested the ideal screw path is parallel of the quadrilateral wall, in which the screw could fix the quadrilateral fracture and prevent the medial subluxation [8]. The placement of this additional screw can also increase the fixation strength of plate for acetabular fractures [4, 5]. On this basis, Culemann et al described the infra-acetabular screw [1]. They showed the operative technique and indication of the screw. However, the placement of the infra-acetabular screw is technically demanding due to the unique and complex anatomy of the osseous area. The knowledge of the correct insertion point and screw direction is essential to avoid penetrating into joint and injury to neurovascular structures [9].

At present, there are multiple studies on the application of CT data utilizing various software for the fixation of periacetabular screws [7, 9–13]. Previous studies have showed that the method of axial perspective can help to find a larger anterior and posterior column screw path [10, 11]. The purpose of the study is to specify the ideal insertion point, the largest secure diameter and length, and the accurate angle of the infra-acetabular screw through the method of axial perspective.

## Materials and methods

We retrospectively collected the pelvic CT scans of 200 adults who had undergone continuous slice CT scanning at the imaging research center of our hospital during August 2016 and June 2019. Patients were excluded if they had pelvic or acetabular fractures, tumors, severe deformities, or severe hip inflammation. This study was approved by the Institutional Review Board of Liaocheng people’s hospital. Due to the retrospective nature of the study, informed consent was waived. The mean age of the patients on whom the models were based was 46.31 ± 14.19 years (range 18–86 years).

DICOM-formatted CT-scan images of each patient were imported into Mimics software (20.0; Materialise, Leuven, Belgium). We removed the soft tissue, femoral head and sacroiliac joint by the function of image segmentation, region growth and multiple slice editing of Mimics software, respectively. A total of 200 right virtual semi-pelvic models were created.

**Fig. 1:**
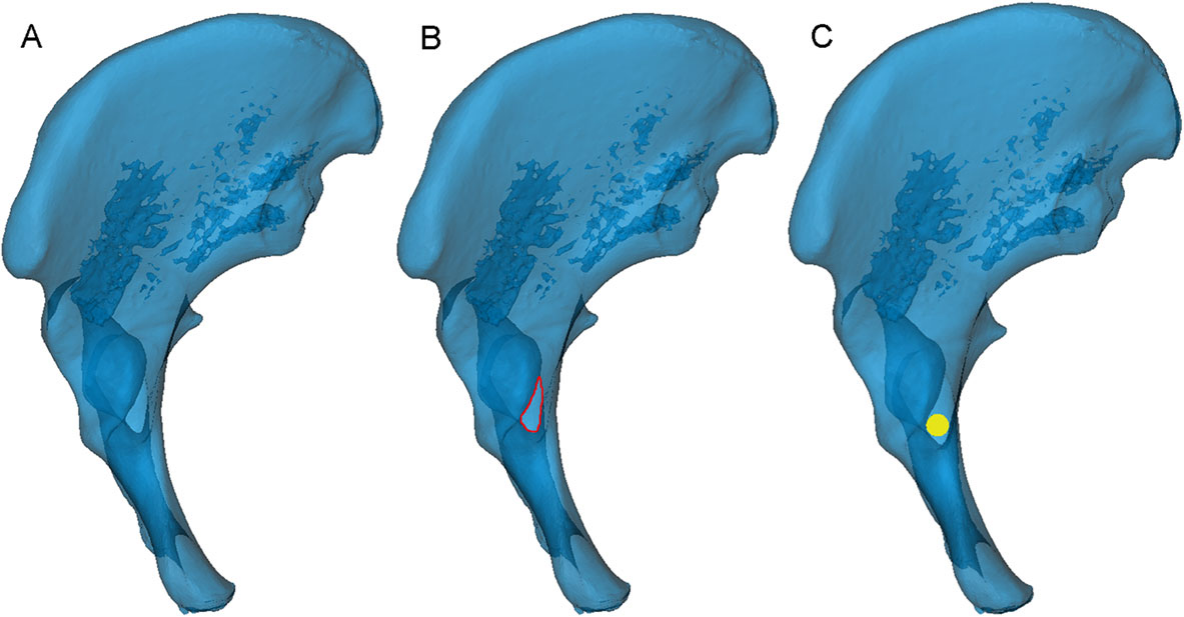
Find the largest screw path. **A** The 3D model was turned to the axial perspective to find the largest translucent area. **B** The outline of this translucent area (marked in red) represented the overlaying cortical bone, like a drop of water. **C** A virtual screw was placed in the centre of the tear-drop area. Then, the diameter was increased progressively until it reached the borderline of the area (the yellow circle of the cylinder represents the largest screw)

**Fig. 2:**
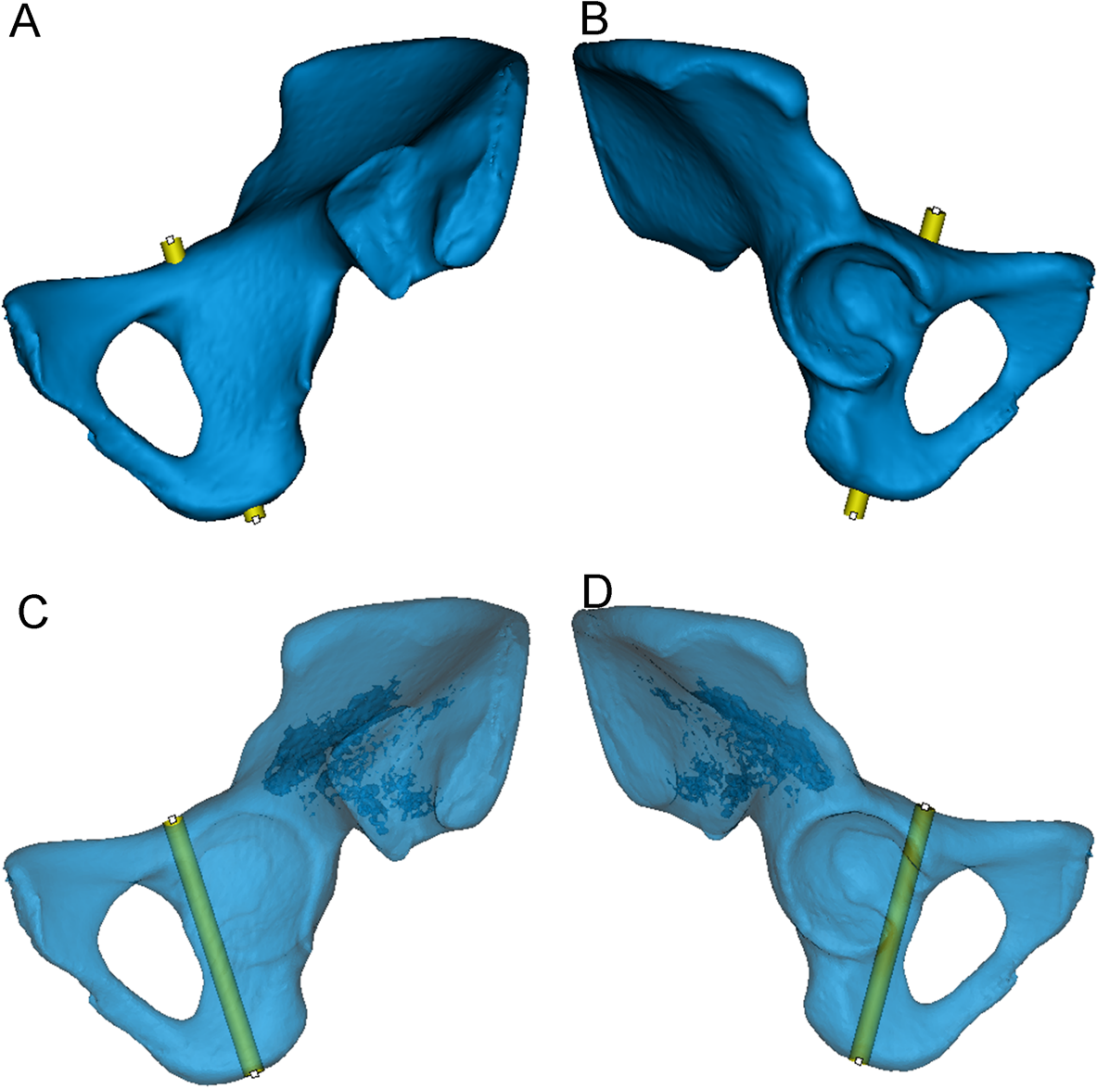
The position of the virtual screw was verified in the 3D model. **A**, **B** Observed from the interior and exterior of the opaque 3D model, respectively. The screw has the largest diameter without penetrating the cortical bone. **C**, **D** Adjusted to the optimal length of the screw from the translucent 3D model

**Fig. 3:**
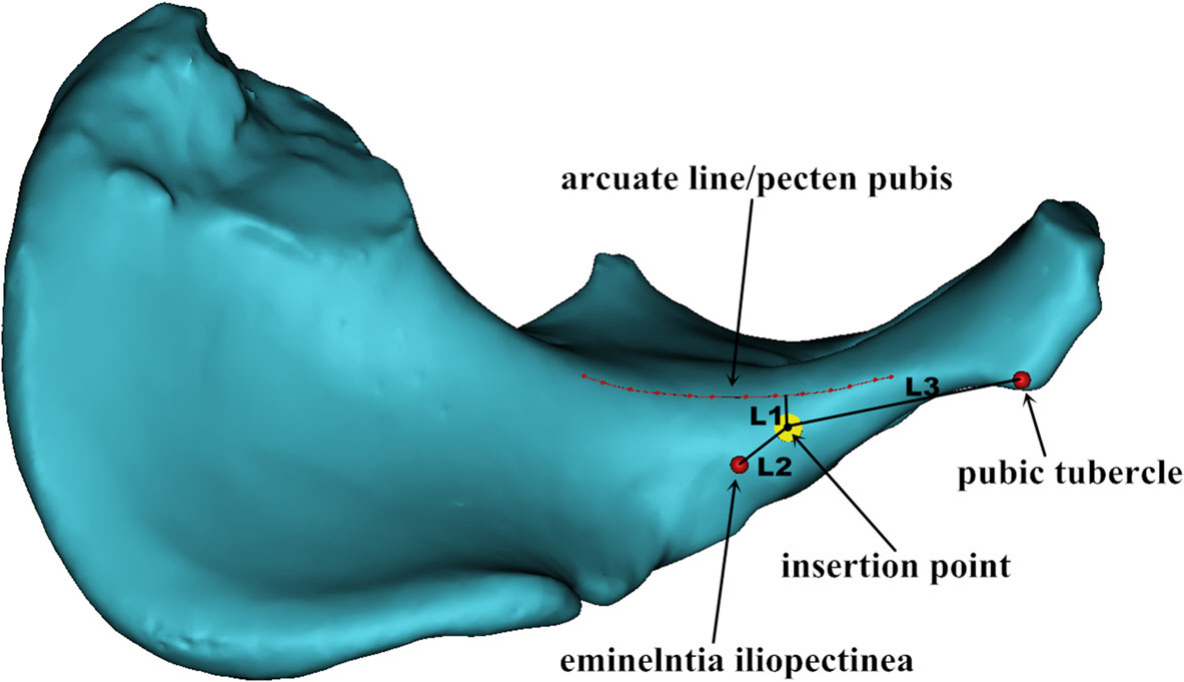
The measurement of Distance L1, L2 and L3. The red curve represented the arcuate line/pecten pubis. The vertical distance from the insertion point to the red curve was recorded as L1. The distances from the insertion point to eminelntia iliopectinea and pubic tubercle were recorded as L2 and L3, respectively

**Fig. 4:**
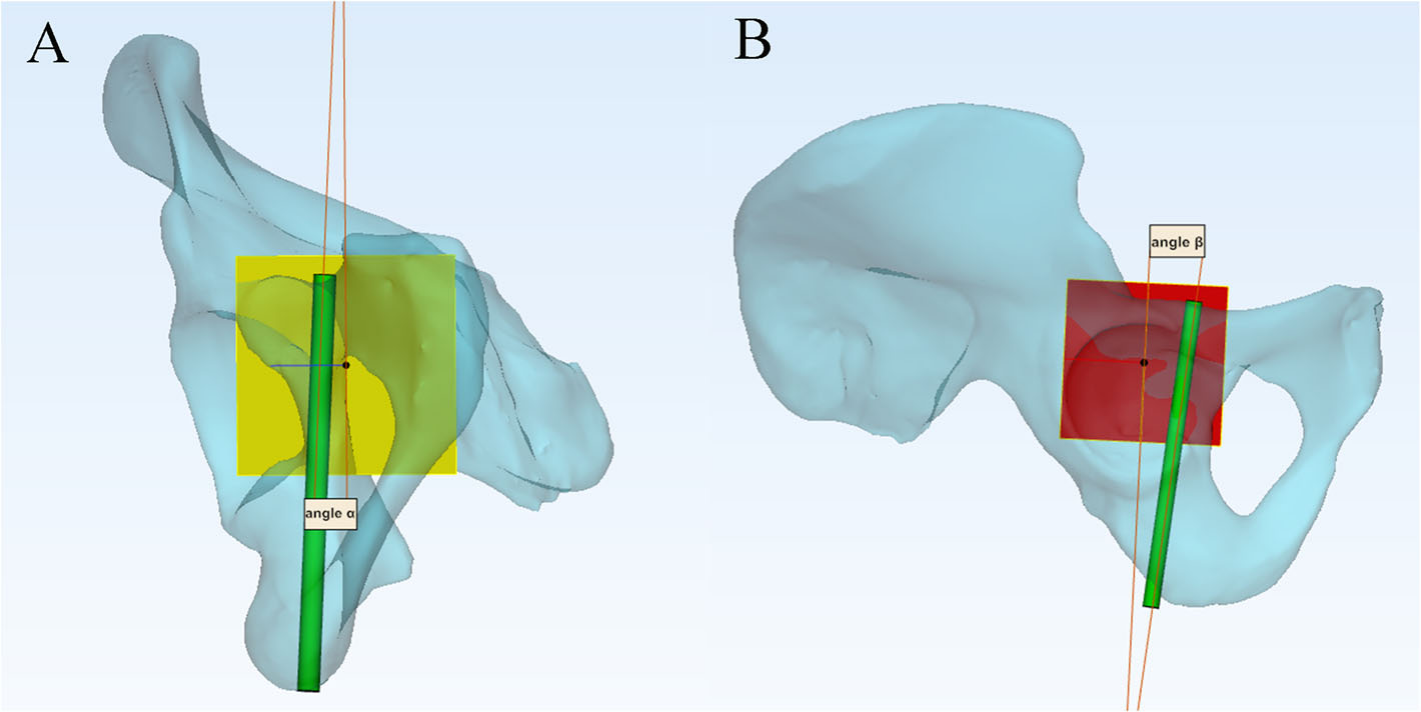
The measurement of Angle α and β. The red plane represented the medial surface of quadrilateral plate. The yellow plane was perpendicular to the red plane. **A** View from the yellow plane, α was the lateral inclination angle between the screw and the red plane. **B** View from the red plane, β was the posterior inclination angle between the screw and the yellow plane

We reduced the transparency of the semi-pelvic models and turned it to the axial perspective view, which was parallel to the posterior of obturator formamen from top to bottom (Fig 1A). Observations and measurements were conducted in different positions of the model through the perspective view to find the largest translucent area. Then, a translucent area like a tear drop was seen clearly (Fig 1B). The area represented the infra-acetabular screw path and the outline represented the medial cortical bone. A virtual cylinder representing the screw was placed into the translucent area. The risk of border penetration was assessed as the diameter of the cylinder increasing progressively and the maximum diameter was defined (Fig 1C). We observed and adjusted the length of the screw to make sure that the screw did not penetrate the cortical bone (Fig 2A–D). The diameter and length of the virtual screw were measured. In order to confirm the position of screw, the distances from the insertion point to the eminelntia iliopectinea, the pubic tubercle and the pelvic border (arcuate line/pecten pubis) were measured, respectively. They were recorded as Distance L1, L2 and L3 (Fig 3). The lateral inclination angle between the screw and the medial surface of quadrilateral plate was measured and recorded as angle α (Fig 4A). In addition, we defined a reference plane perpendicular to the medial surface of quadrilateral plate. The posterior inclination angle between the screw and the reference plane was also measured and recorded as angle β (Fig 4B).

The collected data were analyzed by SPSS 19.0 statistical software. The experimental data are represented as the mean ± SD. T tests were used to compare the data. Statistical significance was accepted at *p* < 0.05.

## Results

The study subjects included 100 males and 100 females aged between 18 and 86 years old, with a mean age of 46.31 ± 14.19 years. As shown in Fig 1B, the screw insertion safe zone exhibited an irregular “tear drop” from the reconstructed pelvic model.

As shown in Tables 1 and 2, the mean maximum diameter of screws was 5.01 ± 1.28 mm and the mean maximum length of screws was 93.99 ± 8.92 mm. The mean distance L1 was 5.99 ± 3.14 mm, L2 was 14.45 ± 3.18 mm, and L3 was 45.81 ± 6.40 mm, respectively. For the data captured above, the intersex difference was significant (*P* < 0.05).

**Table 1:** Comparison between different genders: Diameters of screws, Lengths of screws

Group	Diameter^b^ (mm)	Length^b^ (mm)
All (*n* = 200)	5.01 ± 1.28	93.99 ± 8.92
Male (*n* = 100)	5.54 ± 1.38	100.23 ± 6.37
Female (*n* = 100)	4.49 ± 0.90	87.74 ± 6.36
*t* value^a^	6.391	13.860
*P* value^a^	0.00	0.00

^a^*t* and *P* are the results of gender comparisons

^b^For the Diameter and Length, intersex difference was significant (*P* < 0.05)

**Table 2:** Comparison between different genders: L1, L2 and L3

Group	L1^b^ (mm)	L2^b^ (mm)	L3^b^ (mm)
All (*n* = 200)	5.99 ± 3.14	14.45 ± 3.18	45.81 ± 6.40
Male (*n* = 100)	7.16 ± 2.81	13.90 ± 2.58	47.14 ± 6.20
Female (*n* = 100)	4.82 ± 3.02	15.00 ± 3.61	44.48 ± 6.35
*t* value^a^	5.683	−2.486	2.999
*P* value^a^	0.00	0.014	0.003

^a^t and P are the results of gender comparisons

^b^For the distance of L1, L2 and L3, intersex difference was significant (*P* < 0.05)

The mean angle α and β of different genders were also recorded in Table 3. The former was −0.09° ± 4.39° and the latter was −1.90° ± 8.88°. However, the results were not statistically significant between males and females (*P* > 0.05).

**Table 3:** Comparison between different genders: Angle α and β

Group	α (°)	β (°)
All (*n* = 200)	−0.09 ± 4.39	−1.90 ± 8.88
Male (*n* = 100)	0.28 ± 4.78	−1.99 ± 9.35
Female (*n* = 100)	−0.46 ± 3.96	−1.81 ± 8.43
*t* value^a^	1.204	−0.147
*P* value^a^	0.230	0.883

^a^*t* and *P* are the results of gender comparisons. For the Angle α, a negative value indicates a medial inclination. For the Angle β, a negative value indicates an anterior inclination

**Fig. 5:**
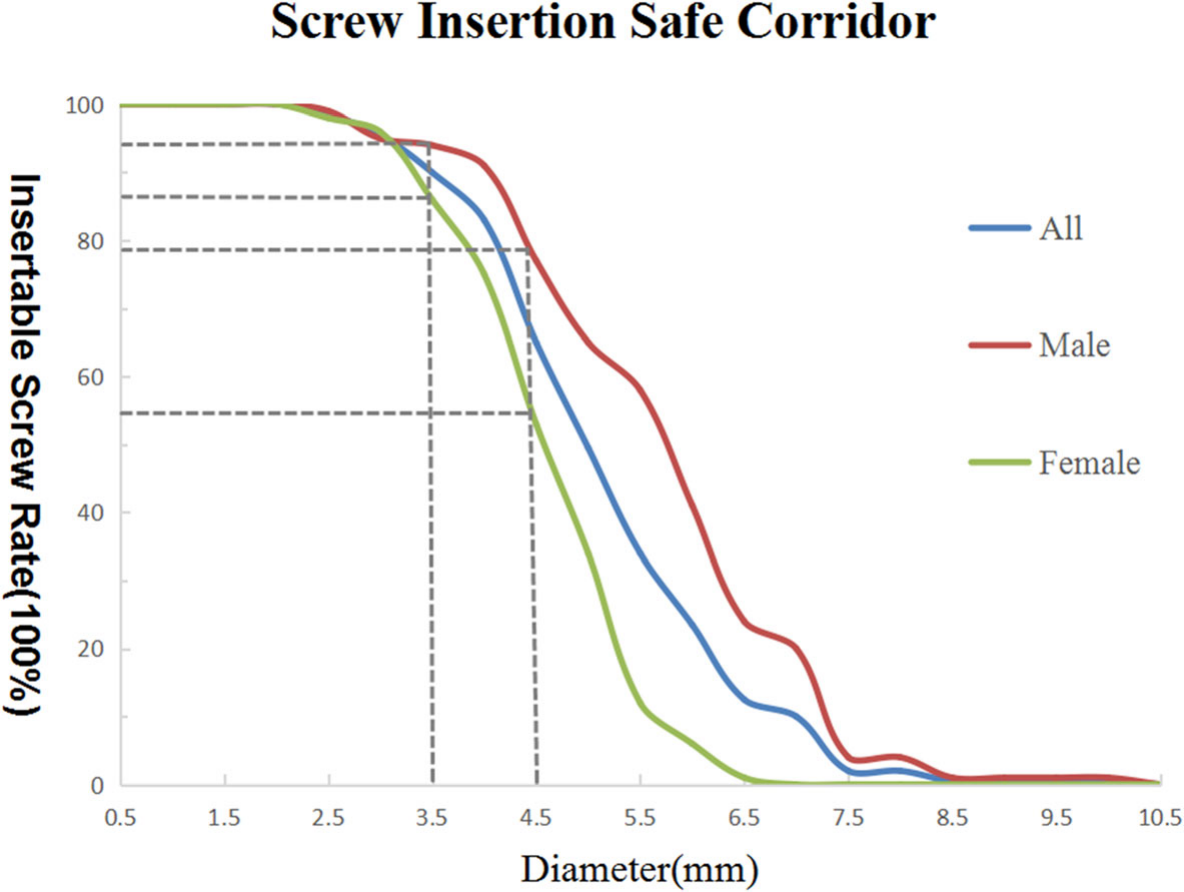
Distribution of the screw insertion safe corridor diameters. The screw insertion corridor with a diameter of at least 3.5 mm was found in 94 of 100 males (94%) and 86 of 100 females (86%). The corridor with a diameter of at least 4.5 mm was found in 77 of 100 males (77%) and 53 of 100 females (53%)

The screw insertion corridor with a diameter of at least 3.5 mm was found in 94 of 100 males (94%) and 86 of 100 females (86%). However, the corridor with a diameter of at least 4.5 mm was found in 77 of 100 males (77%) and 53 of 100 females (53%) from the Fig 5.

## Discussion

In recent years, the trend in treatment of acetabular fractures is achievement of less invasive single ilioinguinal approach, especially in elderly patients [14–18]. Due to the complex characteristic of pelvic anatomy, the safe region of screw placement is far away from the acetabulum, which will reduce the peri-acetabular stability [7]. The common fixation methods for acetabular fractures are positional screw fixation and plate osteosynthesis [10]. Past researches of positional screw fixation have achieved good outcomes [19, 20]. The infra-acetabular screw can be applied via a single ilioinguinal approach to treat acetabular fractures involving a fracture line descending along the acetabular fossa and reaching the obturator formamen [1]. So far, there is no literature that identifies this screw as a positioning screw for the posterior column, and few digital anatomical studies are conducted on its properties.

Mimics software has been widely used in 3D reconstruction for the development of digital orthopedics technology. In our study, we applied the 3D method of axial perspective that described in previous studies were applied to simulate the surgical procedure [10, 11, 21]. We found the largest secure screw path along the longitudinal axis of the anterior part of posterior column after reducing the transparency of the 3D model. Compared with previous studies of computer-assisted determination or virtual three-dimensional model [12, 22], the method of axial perspective showed another osseous channel for positional screw of posterior column. We increased the diameter of virtual cylinder progressively and monitored the virtual screw in the views of coronal plane, sagittal plane and horizontal plane, without violating the cortices and articular surface. Compared with previous human cadaveric studies [23, 24], our method greatly saved manpower, material and financial resources, and can be repeated and verified through highly reliable test results.

In our research, the diameter and length of the infra-acetabular screw were significantly larger in males compared with females. In addition, the vertical distance from the insertion point to the arcuate line or pecten pubis and the distances from the insertion point to eminelntia iliopectinea and pubic tubercle were all measured in this study, which exhibited significant difference between genders. This was due to the obvious anatomic differences in pelvic bones between female and male. This study showed that the mean angle α and β between male and female had no statistical inference. From the Table 3, both angles were close to 0 degrees. This means that the screw is almost parallel to the medial surface of quadrilateral plate and perpendicular to the arcuate line.

Gras et al found that 93% pelves contained an infra-acetabular corridor with a diameter of at least 5 mm [13]. They also provided reference values for placement of a 3.5-mm cortical screw in the corridor. However, in our study, we found that the containable diameter of the screw was smaller in Chinese patients, especially in female. According to the data in our study, the maximum diameter to avoid cortical breaches is 5.54 ± 1.38 mm in male and 4.49 ± 0.90 mm in female. The screw insertion corridor with a diameter of at least 3.5 mm was found in 94 of 100 males (94%) and 86 of 100 females (86%). Only 77 males (77%) and 53 females (53%) possessed a corridor with diameter of at least 4.5 mm as shown in Fig 5. If a positional screw is to be used, a 3.5-mm cortical screw is the first choice and a 4.5 mm-hollow screw may be considered in males. Nevertheless, due to individual and sex differences, the use of preoperative measurements and calculations by digital tools is recommended. Technically, to avoid joint violation, it is crucial to save the subcondral bone while trying to place a juxta articular screw, which requires the use of screws with a smaller diameter than calculated by the preoperative CT scan. The screw diameter also dependents on some other variables like the quality of the reduction and the direction of the screw, so surgeons should prepare different size of screws before the surgery.

On the basis of mastering the diameter and length of screw, the insertion point and direction are important factors affecting the safe placement of infra-acetabular screw. Unlike the common posterior column screw, the infra-acetabular screw needs to be placed through the middle window of ilioinguinal approach. Culemann et al reported that the entry point for the infra-acetabular screw was 1 cm caudal of the eminelntia iliopectinea and in the middle of the pubic ramus [1]. Baumann et al found that the ideal entry point for the infra-acetabular screw was 10.2 mm caudal and 10.4 mm medial of the eminelntia iliopectinea [7]. Gras et al found that the optimized entry points of infra-acetabular screws were located in the mediocaudal region of the eminelntia iliopectinea [13]. Different from previous studies, we found that the optimized insertion point was 13.90 ± 2.58 mm away from the eminelntia iliopectinea in males and 15.00 ± 3.61 mm in females. This is due to the different pelvic shapes between female and male. The anatomic landmark of eminelntia iliopectinea and pubic tubercle can be used as effective references intraoperatively as they are large bony bumps that can be well palpable and identified. The parameters of the infra-acetabular screw may provide the surgeon appropriate information of safe positional screw placement for the treatment of acetabular fracture with separation of both columns. The large standard deviation of our results indicates great differences among individuals. As a result, preoperative planning should be implemented detailedly for each patient. 3D reconstruction and simulated screw placement technique with digital software before operation are valuable.

There are still some limitations to this study. We only analyzed the data based on genders, not different age groups. In addition, we did not collect data according to age, height, weight or body bone density. These factors may also affect the implantation of screws. We only studied the pelvises of Chinese people, who have different skeletal shapes with American and European populations. Thus, more biomechanical studies and related clinical research should be performed to compare the effect of the infra-acetabular screw with other acetabular screws.

## Conclusion

We indicate a valuable guideline for seeking the largest secure corridor of infra-acetabular screw, which is used as a special posterior column positional screw. The ideal screw position and the size of the screws can be determined in 3D-models by digital software. Further biomechanical studies are needed to verify the strength and effect of the screw.

## Data Availability

The datasets generated and analyzed during the current study are available from the corresponding author on reasonable request.

## Abbreviations

3-D: Three-dimensional
CT: Computed tomography
DICOM: Digital Imaging and Communication in Medicine
Mimics: Materialise’s Interactive Medical Image Control System
SPSS: Statistical Package for the Social Sciences
SD: Standard deviation
